# Cdkal1, a type 2 diabetes susceptibility gene, regulates mitochondrial function in adipose tissue

**DOI:** 10.1016/j.molmet.2017.07.013

**Published:** 2017-07-31

**Authors:** Colin J. Palmer, Raphael J. Bruckner, Joao A. Paulo, Lawrence Kazak, Jonathan Z. Long, Amir I. Mina, Zhaoming Deng, Katherine B. LeClair, Jessica A. Hall, Shangyu Hong, Peter-James H. Zushin, Kyle L. Smith, Steven P. Gygi, Susan Hagen, David E. Cohen, Alexander S. Banks

**Affiliations:** 1 Division of Endocrinology, Diabetes and Hypertension, Brigham and Women's Hospital, Boston, MA 02115, USA; 2 Department of Cell Biology, Harvard Medical School, Boston, MA 02115, USA; 3 Dana-Farber Cancer Institute, Boston, MA 02115, USA; 4 Department of Surgery, Beth Israel Deaconess Medical Center, Boston, MA 02115, USA; 5 Division of Gastroenterology & Hepatology, Weill Cornell Medical College, New York, NY 10065, USA

**Keywords:** Cdkal1, GWAS, Diabetes, Adipose, Mitochondria, ANT1, CDKAL1, CDK5 regulatory subunit associated protein 1 like 1, CDK5RAP1, CDK5 regulatory subunit associated protein 1, HFD, high-fat diet, A-KO, adipose-specific Cdkal1 KO, OCR, Oxygen consumption rate, Lys, lysine

## Abstract

**Objectives:**

Understanding how loci identified by genome wide association studies (GWAS) contribute to pathogenesis requires new mechanistic insights. Variants within *CDKAL1* are strongly linked to an increased risk of developing type 2 diabetes and obesity. Investigations in mouse models have focused on the function of *Cdkal1* as a tRNA^Lys^ modifier and downstream effects of *Cdkal1* loss on pro-insulin translational fidelity in pancreatic β−cells. However, *Cdkal1* is broadly expressed in other metabolically relevant tissues, including adipose tissue. In addition, the *Cdkal1* homolog *Cdk5rap1* regulates mitochondrial protein translation and mitochondrial function in skeletal muscle. We tested whether adipocyte-specific *Cdkal1* deletion alters systemic glucose homeostasis or adipose mitochondrial function independently of its effects on pro-insulin translation and insulin secretion.

**Methods:**

We measured mRNA levels of type 2 diabetes GWAS genes, including *Cdkal1*, in adipose tissue from lean and obese mice. We then established a mouse model with adipocyte-specific *Cdkal1* deletion. We examined the effects of adipose *Cdkal1* deletion using indirect calorimetry on mice during a cold temperature challenge, as well as by measuring cellular and mitochondrial respiration *in vitro*. We also examined brown adipose tissue (BAT) mitochondrial morphology by electron microscopy. Utilizing co-immunoprecipitation followed by mass spectrometry, we performed interaction mapping to identify new CDKAL1 binding partners. Furthermore, we tested whether *Cdkal1* loss in adipose tissue affects total protein levels or accurate Lys incorporation by tRNA^Lys^ using quantitative mass spectrometry.

**Results:**

We found that *Cdkal1* mRNA levels are reduced in adipose tissue of obese mice. Using adipose-specific *Cdkal1* KO mice (A-KO), we demonstrated that mitochondrial function is impaired in primary differentiated brown adipocytes and in isolated mitochondria from A-KO brown adipose tissue. A-KO mice displayed decreased energy expenditure during 4 °C cold challenge. Furthermore, mitochondrial morphology was highly abnormal in A-KO BAT. Surprisingly, we found that lysine codon representation was unchanged in *Cdkal1* A-KO adipose tissue. We identified novel protein interactors of CDKAL1, including SLC25A4/ANT1, an inner mitochondrial membrane ADP/ATP translocator. ANT proteins can account for the UCP1-independent basal proton leak in BAT mitochondria. *Cdkal1* A-KO mice had increased ANT1 protein levels in their white adipose tissue.

**Conclusions:**

Cdkal1 is necessary for normal mitochondrial morphology and function in adipose tissue. These results suggest that the type 2 diabetes susceptibility gene *CDKAL1* has novel functions in regulating mitochondrial activity.

## Highlights

• Cdkal1 is a gene most strongly expressed in tissues with high mitochondrial content.
• Cdkal1 is required for normal mitochondrial morphology and function.
• Deletion of Cdkal1 in adipose tissue impairs the thermogenic response to a cold challenge.
• Cdkal1 interacts with ANT1, a mitochondrial ATP/ADP transporter.
• Loss of Cdkal1 does not affect protein translation as predicted for a tRNA modifying enzyme.

## 1 Introduction

Translating results from genome wide association studies (GWAS) into understanding of disease pathogenesis is often hindered by three critical bottlenecks: identifying the genes affected by noncoding variants, ascertaining the tissues affected, and characterizing the molecular function of poorly characterized genes. The *CDKAL1* locus, implicated in type 2 diabetes, is a perfect example of these issues. Polymorphic variants within the *CDKAL1* locus are strongly associated with increased risk of developing type 2 diabetes by GWAS and dozens of replication studies in diverse populations [1], [2], [3], [4], [5], [6], [7]. However, the mechanism linking non-coding variants within *CDKAL1* with diabetes susceptibility remains unclear. Because the disease-associated single nucleotide polymorphisms (SNP) fall within an intronic region of the 700 kb *CDKAL1* locus, in a region sparsely populated by genes, most studies have assumed these SNPs cause changes in *CDKAL1* gene expression. In non-diabetic subjects, *CDKAL1* disease-associated SNPs correlate with impaired insulin secretion, suggesting that β-cells in pancreatic islets may be disproportionately affected [8], [9]. Impaired insulin secretion was similarly observed in *CDKAL1*^−/−^ human ESC differentiated into β cells [10]. Consequently, prior studies on Cdkal1 have focused on *Cdkal1* loss in pancreatic islets and its effects on insulin secretion, although defects in liver, muscle, or adipose tissue, among other tissues, may also impinge on β-cell function [11], [12].

Phenotypes in *Cdkal1* deficient mice are attributed to Cdkal1 function in regulating a specific modification of the cytoplasmic tRNA^Lys(UUU)^. Chemical modifications of tRNAs are an ancient, evolutionarily conserved mechanism to maintain the accuracy of codon recognition [13]. CDKAL1, along with its homolog CDK5RAP1, shares protein domain architecture and 18–23% amino acid identity with the bacterial methyl-thiol transferase (MTT) proteins MiaB, MtaB, and RimO. MTT enzymes utilize two [4Fe-4S] cluster cofactors, bound to an N-terminal MTT domain and a central radical S-adenosyl methionine (SAM) domain, to add a methylthiol moiety (-SCH_3_) to a non-activated carbon on their substrates [14]. Their C-terminal TRAM domains confer substrate specificity. In *Escherichia coli*, RimO is also capable of transferring a methyl-thiol group onto ribosomal protein S12, a post-translational modification not found in eukaryotes [15]. Prokaryotic MtaB and MiaB complete the hyper-modification of tRNA adjacent to the anticodon, a reaction thought to enhance base pairing between RNA codons and associated tRNAs [14], [16]. MtaB performs the final modification of threonylcarbamoyl-modified adenosine (t^6^A_37_) to 2-methylthio-N^6^-threonylcarbamoyl adenosine (ms^2^t^6^A_37_), affecting anticodon pairing with ANN codons [17]. MTT proteins are extensively implicated in affecting protein translation at the ribosome [14]. Cdkal1 in mammals is annotated as a t^6^A_37_ MTT, and decreased levels of the associated ms^2^t^6^A_37_ modification are found in *Cdkal1* knockout mice [18]. Dysfunctional translation of critical Lys residues in pro-insulin and ER stress are reported to cause the impaired insulin secretion from β-cells observed in mice lacking *Cdkal1* [18].

Mice with whole-body *Cdkal1* deletion have been previously described and were observed to be developmentally and phenotypically normal on a standard diet, a finding which is seemingly at odds with *Cdkal1* as a generalized guardian of Lys translational fidelity [19]. These mice exhibit mild impairment of glucose tolerance when challenged with 20 weeks on a high fat diet (HFD) [19], [20]. Mice with pancreatic β-cell specific *Cdkal1* knockout have also been previously characterized. Those mice displayed a more pronounced phenotype, with strongly impaired glucose tolerance on both normal chow and HFD, a finding attributed to defects in pro-insulin translation [18].

Here we investigate the biological role of Cdkal1 in adipose tissue *in vivo* using a mouse model with adipocyte-specific knockout (A-KO) of *Cdkal1*. Mice lacking adipose *Cdkal1* exhibit features of impaired brown adipose tissue (BAT) mitochondrial function including decreased energy expenditure when challenged with cold temperature. Primary brown adipocytes differentiated *ex vivo* from digested *Cdkal1* knockout BAT had decreased rates of respiration. In addition, electron micrographs of *Cdkal1* A-KO BAT revealed highly disrupted mitochondrial morphology, and respiration experiments in isolated A-KO BAT mitochondria confirmed functional defects. Interestingly, we did not observe differences in Lys codon utilization by mass spectrometry in adipose tissue, as would be predicted based on its function as tRNA modifier, nor were differences in glucose homeostasis apparent in *Cdkal1* A-KO mice. To identify potential novel functions for CDKAL1 independent of tRNA^Lys^ modification, we performed unbiased protein interaction mapping to find new CDKAL1 binding partners. Through these studies, we identified the interaction between CDKAL1 and ANT1/Slc25a4, the mitochondrial adenine nucleotide translocator protein, which may provide a mechanistic link between CDKAL1 and mitochondrial dysfunction. Taken together, these findings in adipose tissue suggest that the type 2 diabetes GWAS candidate gene *Cdkal1* has a functional role in regulating mitochondrial function *in vivo*.

## 2 Materials & methods

### 2.1 Animals

Mice with a conditional allele of *Cdkal1* (*Cdkal1*^flox^) were generated by crossing the “null-first” mice Cdkal1^tm2a(EUCOMM)Wtsi^ (Emma European Mouse Mutant Archive) to mice expressing enhanced FLP1 recombinase (Jackson Labs, 005703) [21]. *Cdkal1*^flox^ mice were bred with adipocyte-specific Adipoq-Cre^1Evdr/J^ [22] (Jackson Labs, 010803) to generate mice with adipocyte-specific deletion of *Cdkal1* (A-KO) (Supplementary Figure 2A). Routine genotyping was performed with the following three primers, 1608 (sense):5′-CTTCTTGTGACTCCTTGGTTA-3′, 1609 (antisense): 5′-CAACGGGTTCTTCTGTTAGTCC-3′, 1610 (antisense):5′-GCTGTCCAGCCATGTATTCTC-3′. The wild-type 1608–1610 amplicon is 670 bp. The 1608–1609 amplicon from the floxed allele is 750 bp. Unless stated otherwise, 8–14 week old male mice were examined against littermates while maintained on a standard chow diet (PicoLab Rodent Diet 20, #5053). Control mice are *Cdkal1*^flox/flox^ genotype, while A-KO mice are Adipoq-Cre^+^::*Cdkal1*^flox/flox^. For chow vs high-fat diet studies in wild type mice, as well as Cdkal1 mRNA tissue distribution, C57BL/6J mice were purchased from Jackson Laboratories (Jackson Labs, 000664). High fat diet with 60% of calories from fat was obtained from Research Diets (Research Diets, #D12492, irradiated). All animal studies were approved by the Brigham and Women's Hospital IACUC.

### 2.2 Indirect calorimetry

Control and A-KO male mice were implanted with temperature probes 5–7 days before the beginning of indirect calorimetry experiments. Mice were maintained on a standard chow diet and housed at thermoneutrality (30 °C) for 48–72 h prior to experiment start. The temperature transition from 30 °C to 4 °C was performed over a period of 3 h. Oxygen consumption, CO_2_ emission, food consumption, movement, and energy expenditure were measured using a 24 cage CLAMS apparatus (Columbus Instruments) available to the Brigham and Women's Hospital Metabolic Phenotyping Core. Mice were implanted with intraperitoneal telemetry temperature probes one week prior to the start of measurement. Statistical analysis and plotting was performed in the R programming language with CalR, a custom package for analysis of indirect calorimetry using analysis of covariance with a graphical user interface.

### 2.3 Glucose tolerance tests and insulin tolerance tests

Diet-induced obesity (DIO) in *Cdkal1* A-KO mice, along with littermate controls, was generated by feeding mice a high fat, high sugar diet (HFD) which delivers 60% of its caloric content as fat (Research Diets, D12492). Mice were established on HFD feeding after weaning, at 3–5 weeks of age. Body mass was monitored weekly. Body composition of DIO mice was measured by EchoMRI at 10–11 weeks of HFD feeding. Intraperitoneal glucose tolerance test (IP-GTT) on HFD-fed mice was conducted after 11–13 weeks on HFD feeding. Mice were given one week to recover after IP-GTT, and were subjected to insulin tolerance testing (ITT) after 12–15 weeks of HFD feeding. Prior to IP-GTT, HFD-fed mice were singly housed and fasted overnight (16 h). HFD-fed mice received 1 g/kg body weight glucose via i.p. injection of appropriate volume of 20% (w/v) glucose solution in Millipure H_2_O. Prior to ITT, mice were singly housed and fasted for 4 h before receiving an i.p. injection of 1.5 U/kg body weight insulin in sterile PBS. After injection, blood glucose levels were monitored by glucometer strip readings of tail bleeds at time points up to 2 h. Normal chow-fed cohorts of A-KO mice were also subjected to IP-GTT and ITT at approximately 11 weeks and 12 weeks post-weaning, respectively. Chow-fed animals were followed for body mass and EchoMRI body composition measurements as described above. Chow-fed A-KO cohorts were singly housed and fasted before assays for the same lengths of time as described above. For IP-GTT, chow-fed mice were injected i.p. with 2 g/kg body weight glucose. For ITT, chow-fed mice received 0.75 U/kg body weight insulin.

### 2.4 Gene expression and qPCR

RNA from tissue samples was isolated from Qiazol (Qiagen) using isopropanol extraction and from TRI reagent using the Direct-Zol RNA miniprep kit (Zymo Research). cDNA was generated from 1 μg RNA by RT-PCR with Multiscribe Reverse Transcriptase and High-capacity cDNA Reverse Transcription Kit (Applied Biosystems). Quantitative PCR was performed using SYBR select Master Mix (Applied Biosystems) on a Light Cycler 480 II (Roche). Expression levels were assessed using the ΔΔCt method, using TBP as a control gene. For GWAS candidate gene analysis in Supplementary Figure 1, genes with a cycle threshold >30 were considered not expressed. The list of primers used in this study is included in Supplementary Table S1.

### 2.5 Western blotting

Tissue fragments were homogenized in lysis buffer containing 50 mM Tris-HCl (pH 7.5), 150 mM NaCl, 1% NP-40, 0.5% sodium deoxycholate, and 0.1% SDS using ball bearings and a TissueLyser (Qiagen). Lysis buffer was supplemented with HALT protease and phosphatase inhibitor cocktail (Thermo Fisher). Protein lysates were quantitated using Pierce BCA protein assay (Thermo Fisher). Western blotting was performed using Miniprotean TGx 4–20% SDS-PAGE gels, Tetra Cell rig, and Transblot Turbo transfer system (Biorad). Membranes were blocked with 5% milk in 1× TBS-T, before overnight incubation with primary antibodies. HRP-conjugated secondary antibodies against rabbit- and mouse-derived primary antibodies were used at 5000-fold dilution and were obtained from Cell Signaling Technologies (7074S, 7076S). Chemiluminescence was visualized and recorded digitally using the Chemidoc XRS + Imaging System (Biorad). Primary antibodies used in this study include: rabbit anti-Cdkal1 (abcam, ab68045), mouse anti-ANT1 (abcam, 110322), rabbit anti-MMS19 (Proteintech, 16015-1-AP), mouse anti-total OXPHOS rodent cocktail (abcam, ab110413), mouse anti-FAM96B (Santa Cruz, sc-376801), mouse anti-FLAG tag (Sigma, F1804), mouse anti-FLAG M2 HRP-conjugated (Sigma, A8592), rabbit anti-UCP1 (abcam, 23841), and rabbit anti-β-Tubulin (Cell Signaling, 2146).

### 2.6 Cellular respiration

Stromal vascular fraction (SVF) was generated from BAT, and primary brown adipocytes were differentiated *in vitro* as described [23]. Briefly, BAT depots were dissected from euthanized 2–3 week old mice. Tissue was digested for 45 min shaking at 37 °C in digestion medium consisting of PBS with 1.3 mM CaCl_2_, 123 mM NaCl, 5 mM KCl, 5 mM d-dextrose, 100 mM HEPES, 4% (w/v) BSA, and 1.5 mg/mL collagenase B (Roche). Digestion reaction was terminated with growth medium consisting of high-glucose DMEM/F12 supplemented with l-glutamine, penicillin/streptomycin, and 10% FBS. Cell suspension was filtered through 70 μm and 40 μm cell strainers. Resuspended SVF cells were plated in growth medium and expanded for 5–7 days. For differentiation for Seahorse assay, cells were plated at defined density (10,000 or 15,000 cells/well) into wells of Seahorse XF24 v7 culture plates (Agilent). The following day, cells were differentiated in growth medium supplemented with 0.02 μM insulin, 1 μM rosiglitazone, 125 μM isobutylmethylxanthine, 2 μg/mL (5 μM) dexamethasone, 1 nM T3, and 125 μM indomethacin. Cells were maintained on differentiation medium for 48 h, then maintained in growth medium supplemented with only 0.02 μM insulin, 1 μM rosiglitazone. Oxygen consumption rates in primary brown adipocytes were measured by Seahorse assay at d5–d7 of differentiation, as described [23]. Cells were washed once with respiration medium (Seahorse XF base medium with 1 mM pyruvate, 20 mM glucose, and 1× pen/strep), supplied with 0.5 mL/well respiration medium, and incubated in CO_2_-less conditions for 45–60 min. Equilibrated Seahorse XFe24 cartridges were loaded with port injections (75 μL/port) to deliver the following drug treatments: oligomycin (1 μM final), FCCP (0.4 μM final), rotenone (3 μM final). Three measurements were made under basal conditions and after each drug injection. Each measurement cycle had these time parameters: mix 4 min, wait 0 min, measure 2 min. Data are presented as average well OCR (pmol O_2_/min) at each time point. Error bars represent SEM. Basal, proton leak, and maximal respiration were computed by subtracting the non-mitochondrial respiration values, represented by the averaged OCR after rotenone injection, from the averaged untreated, oligomycin injected, and FCCP injected OCR, respectively.

### 2.7 Isolated mitochondria respiration

Brown adipose tissue mitochondria were isolated from 23 to 24 week old, chow-fed male *Cdkal1* adipose knockout mice (n = 10), along with Adipoq-Cre^−^ littermate controls (n = 10) as previously described [24]. Following isolation, mitochondria were kept at 4 °C in storage buffer (100 mM KCl, 20 mM K+ TES, pH 7.2) until aliquots were taken for respiration assay, or for frozen storage (in 50 μg or 200 μg aliquots) at −80 °C. Protein concentration of isolated mitochondria was measured using Pierce BCA protein assay (Thermo Fisher). BAT mitochondria were loaded into Seahorse XF24 V7 culture microplates (Agilent), with 15 μg mitochondria protein in 50 μL respiration medium (125 mM sucrose, 20 mM K+ TES pH 7.2, 2 mM MgCl_2_, 1 mM EDTA, 4 mM KH_2_PO_4_, 0.1% fatty acid-free BSA, 10 mM pyruvate, 5 mM malate) per well. Mitochondria were spun onto the plate at 2000×G for 20 min at 4 °C. Equilibrated Seahorse XFe24 cartridges were loaded with port injections (75 μL/port) to deliver the following drug treatments: GDP (1 mM final), oligomycin (14 μM final), FCCP (10 μM final), rotenone (20 μM final).

### 2.8 Electron microscopy

BAT was fixed by vascular perfusion in 2% glutaraldehyde/0.1M cacodylate buffer, pH 7.4, excised, and fixed for 30 min at RT in the same buffer. Tissues were then cut into small pieces, washed in cacodylate buffer, and frozen in a high pressure freezer (Wohlwend Compact 02 High Pressure Freezer, Technotrade International, Manchester NH, USA). Tissues were processed for resin embedding using the super-quick freeze substitution technique [25], [26]. Ultrathin sections were cut with a Leica Ultracut E ultramicrotome (Leica Microsystems, Wetzlar, Germany), placed on formvar and carbon-coated grids, and examined in a JEOL 1400 electron microscope (JEOL, USA, Peabody, MA, USA) equipped with a Gatan (Pleasanton, CA, USA) Orius CCD camera.

### 2.9 TMT mass spectrometry of total peptides from visceral adipose tissue

Peptides for tandem mass tag labeling mass spectrometry (TMT MS) were isolated from frozen whole visceral white adipose tissue depots isolated from 16 week old Ctrl (n = 5) and A-KO (n = 5) chow-fed mice. TMT reagents (0.8 mg) were dissolved in anhydrous acetonitrile (40 μL) of which 10 μL was added to the peptides (100 μg) along with 30 μL of acetonitrile to achieve a final acetonitrile concentration of approximately 30% (v/v). Following incubation at room temperature for 1 h, the reaction was quenched with hydroxylamine to a final concentration of 0.3% (v/v). The TMT-labeled samples were pooled at a 1:1 ratio across the 10 samples. The pooled sample was vacuum centrifuged to near dryness and subjected to C18 solid-phase extraction (SPE) (Sep-Pak, Waters). Next, we fractionated the pooled TMT-labeled peptide sample using BPRP HPLC [27]. We used an Agilent 1100 pump equipped with a degasser and a photodiode array (PDA) detector (set at 220 and 280 nm wavelength) from Thermo Fisher Scientific (Waltham, MA). Peptides were subjected to a 50-min linear gradient from 5% to 35% acetonitrile in 10 mM ammonium bicarbonate pH 8 at a flow rate of 0.6 mL/min over an Agilent 300 Extend C18 column (3.5 μm particles, 4.6 mm ID and 220 mm in length). The peptide mixture was fractionated into a total of 96 fractions, which were consolidated into 12. Samples were acidified with 1% formic acid and vacuum centrifuged to near dryness. Each consolidated fraction was desalted via StageTip, dried again via vacuum centrifugation, and reconstituted in 5% acetonitrile, 5% formic acid for LC-MS/MS processing. All samples were analyzed on an Orbitrap Fusion Lumos mass spectrometer (Thermo Fisher) coupled with a Proxeon EASY-nLC 1000 liquid chromatography (LC) pump (Thermo Fisher). Peptides were separated on a 100 μm inner diameter microcapillary column packed with 35 cm of Accucore C18 resin (2.6 μm, 150 Å, Thermo Fisher). For each analysis, we loaded approximately 2 μg onto the column. Peptides were separated using either a 180 min gradient of 3–25% acetonitrile in 0.125% formic acid with a flow rate of 500 nL/min. Each analysis used an MS3-based TMT method [28], [29], which has been shown to reduce ion interference compared to MS2 quantification [30]. The scan sequence began with an MS1 spectrum (Orbitrap analysis, resolution 120,000, 400–1400 Th, automatic gain control (AGC) target 5E5, maximum injection time 100 ms). The top ten precursors were then selected for MS2/MS3 analysis. MS2 analysis consisted of collision-induced dissociation (CID), quadrupole ion trap analysis, automatic gain control (AGC) 8E3, NCE (normalized collision energy) 35, q-value 0.25, maximum injection time 150 ms, isolation window at 0.7 Th. Following acquisition of each MS2 spectrum, we collected an MS3 spectrum using a recently described method in which multiple MS2 fragment ions are captured in the MS3 precursor population using isolation waveforms with multiple frequency notches [22]. MS3 precursors were fragmented by HCD and analyzed using the Orbitrap (NCE 55, AGC 1E5, maximum injection time 150 ms, resolution was 50,000 at 400 Th) and an isolation window of 2 Th.

### 2.10 Analysis of TMT MS data and Lys codon utilization

Mass spectra were processed using a Sequest-based in-house software pipeline [31]. Spectra were converted to mzXML using a modified version of ReAdW.exe. Database searching included all entries from the human UniProt database. This database was concatenated with one composed of all protein sequences in the reversed order. Searches were performed using a 50 ppm precursor ion tolerance for total protein level analysis. The product ion tolerance was set to 0.9 Da. These wide mass tolerance windows were chosen to maximize sensitivity in conjunction with Sequest searches and linear discriminant analysis [31], [32]. TMT tags on lysine residues and peptide N termini (+229.163 Da) and carbamidomethylation of cysteine residues (+57.021 Da) were set as static modifications, while oxidation of methionine residues (+15.995 Da) was set as a variable modification. Peptide-spectrum matches (PSMs) were adjusted to a 1% false discovery rate (FDR) [33], [34]. PSM filtering was performed using a linear discriminant analysis, as described previously [31], while considering the following parameters: XCorr, ΔCn, missed cleavages, peptide length, charge state, and precursor mass accuracy. For TMT-based reporter ion quantitation, we extracted the summed signal-to-noise (S:N) ratio for each TMT channel and found the closest matching centroid to the expected mass of the TMT reporter ion. For protein-level comparisons, PSMs were identified, quantified, and collapsed to a 1% peptide false discovery rate (FDR) and then collapsed further to a final protein-level FDR of 1%. Moreover, protein assembly was guided by principles of parsimony to produce the smallest set of proteins necessary to account for all observed peptides. Proteins were quantified by summing reporter ion counts across all matching PSMs using in-house software, as described previously [31]. PSMs with poor quality, MS3 spectra with more than eight TMT reporter ion channels missing, MS3 spectra with TMT reporter summed signal-to-noise ratio that were less than 100, or had no MS3 spectra were excluded from quantification [35]. For each identified peptide, the corresponding cDNA sequence was queried using the UniProt.ws package in the R statistical programming language [26], [36], [37]. The peptide abundance and frequency was assessed for each mouse and for the AAA-Lys codon and for all other codons. We also examined the subset of peptides with abundance more than 2-fold downregulated in A-KO adipose tissue for differences in codon frequency.

### 2.11 Identification of CDKAL1 interacting proteins by co-immunoprecipitation and mass spectrometry

Transfection of HEK293 cells with FLAG-tagged CDKAL1 constructs, immunoprecipitation, and mass spectrometry were performed as in the BioPlex project [25], [38] with data analysis performed as described in COMPASS [39].

### 2.12 Mitochondrial DNA content

Relative mtDNA content in control and *Cdkal1* A-KO BAT was measured using qPCR as described previously [40]. Genomic DNA was extracted from BAT snippets using the DNeasy Blood & Tissue kit (Qiagen). For each sample, 20 ng of genomic DNA were subjected to qPCR with primers for *CytB* (Fwd: GCTTTCCACTTCATCTTACCATTT; Rev: TGTTGGGTTGTTTGATCCTG) and *β-actin* (Fwd: GGAAAAGAGCCTCAGGGCAT; Rev: GAAGAGCTATGAGCTGCCTGA), to measure mtDNA and nuclear DNA content, respectively. Mt/N DNA ratio was calculated using the ΔΔCt method, and data were normalized to average control Mt/N.

### 2.13 Blue native PAGE and Coomassie stain of mitochondrial complexes

Frozen pellets of 200 μg BAT mitochondria were lysed in 100 μL of NativePAGE sample buffer, supplemented with 1× HALT protease and phosphatase inhibitor cocktail and 1% n-dodecyl-β-D-maltoside (DDM), using a NativePAGE Sample Prep kit (Thermo Fisher). Aliquots of 20 μg lysed mitochondria were supplemented with Coomassie G-250 to final concentration of 0.25%, then run out on native 4–16% bis–tris gels using the Native PAGE gel system (Thermo Fisher). Gels were treated with Fix Solution (40% methanol, 10% acetic acid), microwaved for 45 s, and destained with 8% acetic acid for up to 5 h before imaging.

### 2.14 Co-immunoprecipitation of FLAG-tagged ANT1 and *Cdkal1*

HEK293 cells were transfected with empty pcDNA 3.1 vector or pcDNA 3.1 vectors expressing FLAG-Slc25a4/ANT1 (Genscript, Clone#OHu22413) or FLAG-tagged mutants of Cdkal1. Transfected cells were harvested and lysed in non-ionic coIP buffer (50 mM Tris HCl, 150 mM NaCl, 1% Triton X100, 1 mM EDTA) supplemented with 1× HALT cocktail (Thermo Fisher). Protein lysate (1.5 mg for FLAG-ANT1; 10 mg for FLAG-Cdkal1 fragments) was incubated with anti-FLAG M2 affinity agarose (Sigma, A220) rotating overnight at 4 °C. To preserve ANT1 protein, FLAG-tagged baits and co-immunoprecipitated proteins were eluted off agarose using 30 min incubation at 37 °C with SDS sample buffer. Western blotting of co-immunoprecipitation eluates was performed as described above.

## 3 Results

### 3.1 Expression of type 2 diabetes GWAS gene *Cdkal1* is negatively regulated in obese mouse adipose tissue

We first examined whether obesity alters gene expression of type 2 diabetes-associated genes in adipose tissue. We examined mRNA expression of 29 type 2 diabetes GWAS genes following 16 weeks of high-fat, high-sugar diet (HFD) or standard chow in wild-type C57Bl/6J mice. In epididymal adipose tissue, obesity affected mRNA levels of 8 genes, including *Cdkal1*. *Cdkal1*, *Cdc123*, *Jazf1*, *Adamts9*, and *Tmem195* had decreased adipose expression levels in obesity (Supplementary Figure 1A), while levels of *Camk1d*, *Hhex*, and *Igf2bp2* were increased (Supplementary Figure 1B). There were no significant differences in mRNA levels for another 12 genes (Supplementary Figure 1C), while mRNA levels were too low to quantify for 9 remaining genes (*Adcy5*, *Bcl11a*, *Gck*, *Gckr*, *Kcnj11*, *Kcnq1*, *Prox1*, *Slc2a2*, *Slc30a8*) (data not shown). *Cdkal1* is also downregulated in inguinal adipose tissue but not liver, suggesting adipose-specific regulation of its expression with obesity (Supplementary Figure 1D). In lean animals, *Cdkal1* is widely expressed with highest levels observed in mitochondria-rich tissues like heart and skeletal muscles (Supplementary Figure 1E). The regulation of Cdkal1 with obesity and the strong association between obesity and type 2 diabetes suggested further investigation into adipose Cdkal1 was warranted.

### 3.2 Adipocyte-specific *Cdkal1* knockout (A-KO) mice have normal body weight, glucose tolerance, and insulin sensitivity in both the lean and obese states

**Figure 1:**
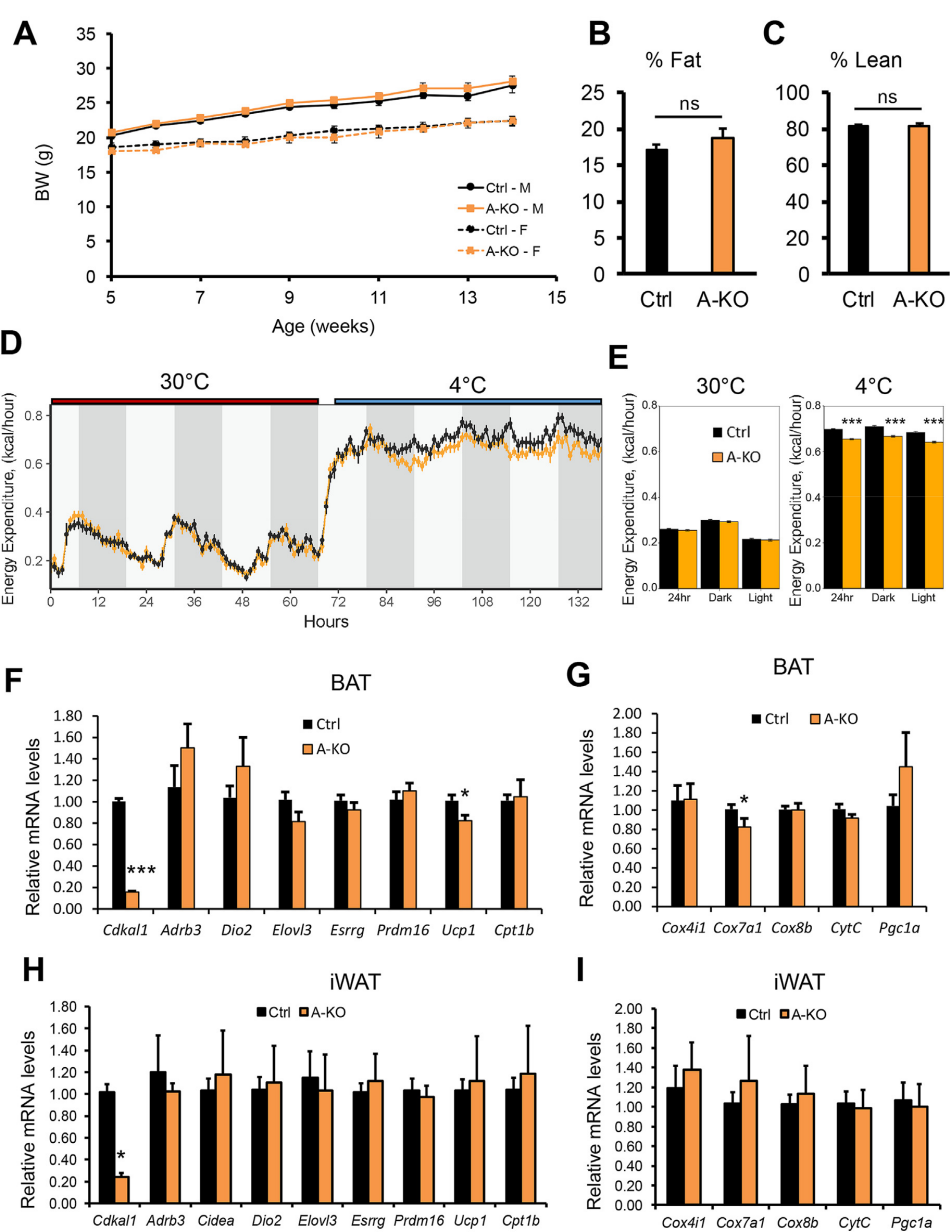
**Effect of adipocyte-specific *Cdkal1* deficiency on cold-induced energy expenditure**. (A) Body weight for male (square) or female (circle) control or *Cdkal1* A-KO mice. Body composition represented as percent body fat mass (B) and percent lean mass (C). (D) Indirect calorimetry measurements of chow-fed Ctrl (n = 6) and *Cdkal1* A-KO (n = 6) mice implanted with an intraperitoneal temperature probe and monitored during cold temperature challenge at 4 °C. Shown are plots of energy expenditure at thermoneutrality (30 °C) and through a cold challenge (4 °C). (E) 24 h and 12 h mean energy expenditure values were unchanged at thermoneutrality but were significantly decreased over combined day/night periods for the duration of the cold challenge by ANCOVA, using total body mass as the co-variate. Error bars represent SEM. ***, p < 0.001. (F) mRNA expression of thermogenic (F) and mitochondrial (G) genes in BAT isolated from Ctrl (n = 8; black bars) and *Cdkal1* A-KO (n = 5; orange bars) mice following 4 °C cold exposure for 3 days. mRNA expression of the same thermogenic (H) and mitochondrial (I) genes was also measured in inguinal WAT isolated from Ctrl (n = 6; black bars) and *Cdkal1* A-KO (n = 6; orange bars) chow-fed male mice following 4 °C cold exposure for 2.5 days. Error bars represent SEM. *, p < 0.05; **, p < 0.01; ***, p < 0.001, Student's *t*-test.

We next asked whether loss of Cdkal1 in adipose tissue specifically affected glucose tolerance in mice. To study the function of Cdkal1 in adipose tissue, we generated mice with adipocyte-specific deletion of *Cdkal1* (Supplementary Figure 2). Mice with a *Cdkal1* conditional allele (*Cdkal1*^*flox*^) were crossed to Adiponectin-Cre mice to generate mice with adipocyte-specific *Cdkal1* ablation (A-KO) (Supplementary Figure 2A). Decreased levels of Cdkal1 protein and mRNA in adipose tissue depots of A-KO mice were confirmed by Western blotting (Supplementary Figure 2B) and qPCR (Supplementary Figure 2C). Mice with adipocyte-specific deletion of *Cdkal1* exhibit similar body weight and body composition relative to *Cdkal1*^*flox/flox*^ controls (Figure 1A–C). Neither glucose (Supplementary Figure 3A) nor insulin tolerance (Supplementary Figure 3B) was significantly altered in *Cdkal1* A-KO mice on a standard chow diet. In addition, adipose-specific deletion of *Cdkal1* did not change glucose tolerance (Supplementary Figure 4A, 4B), insulin tolerance (Supplementary Figure 4C), body weight (Supplementary Figure 4D, 4E) or body composition (Supplementary Figure 4F) in mice rendered obese by HFD feeding. These findings demonstrate that adipose Cdkal1 loss is not sufficient to affect whole body energy balance or glucose homeostasis.

### 3.3 Reduced energy expenditure in *Cdkal1* A-KO mice during cold-temperature thermogenic challenge

Because we observed *Cdkal1* expression to be highest in mitochondria-rich tissues (Supplementary Figure 1E), we next tested whether adipocyte deletion of *Cdkal1* affected function of mitochondria-rich brown adipose tissue (BAT). We monitored control and A-KO male mice by indirect calorimetry during thermoneutrality (30 °C) and during a cold challenge (4 °C). BAT is inactive while mice are maintained at thermoneutrality and, accordingly, there are no observed differences in energy expenditure at 30 °C (Figure 1D and E). However, shortly after initiation of cold challenge, A-KO mice have decreased energy expenditure which was evident during both the light and dark phases (Figure 1D and E). Although energy expenditure was reduced in A-KO mice during the thermogenic response at 4 °C, we did not observe a significant reduction in body temperature in A-KO over the duration of the cold challenge (data not shown). To understand this diminished metabolic rate, we examined mRNA levels of thermogenic and browning markers in BAT (Figure 1F and G) and inguinal subcutaneous adipose tissue (iWAT) (Figure 1H and I) isolated from chow-fed A-KO mice following 4 °C cold challenge. In BAT, the uncoupling protein, *Ucp1* levels were decreased by 20%, an effect not seen in iWAT. Similarly, we examined mRNA levels of genes encoding mitochondrial or mitochondria biogenesis proteins. We found that levels of the nuclear-encoded mitochondrial cytochrome c oxidase subunit 7A1 (*Cox7a1*) mRNA were mildly but significantly decreased in *Cdkal1* A-KO BAT but not iWAT following 4 °C cold challenge. However, mRNA levels of all other thermogenic and mitochondrial genes tested were unchanged in both BAT and iWAT of cold-challenged *Cdkal1* A-KO mice. These data suggest that the decreased *in vivo* response to a cold challenge was not mediated by large transcriptional differences in mRNA levels of thermogenic and mitochondrial genes.

### 3.4 Impaired mitochondrial respiration measured by Seahorse *in vitro* in primary brown adipocytes and isolated BAT mitochondria lacking Cdkal1

**Figure 2:**
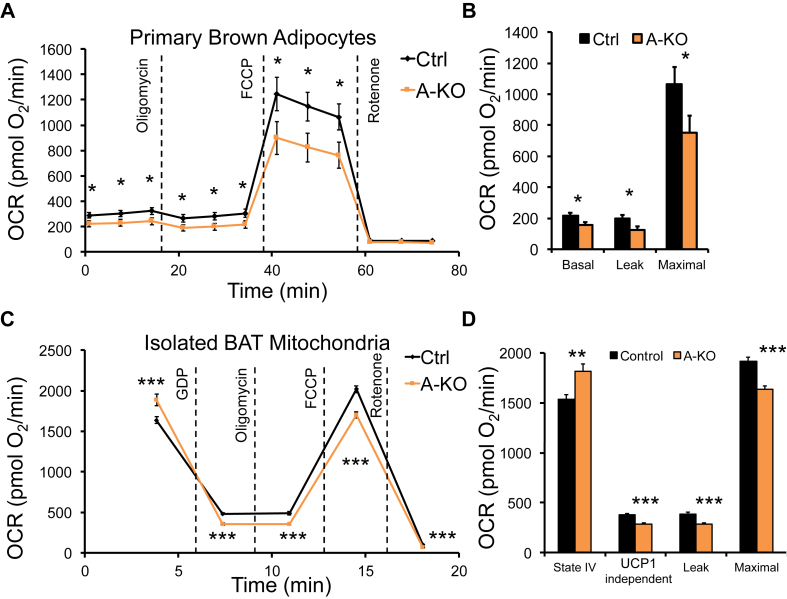
**Cdkal1 is required for normal adipose cellular and mitochondrial respiration**. (A) Oxygen consumption rate (OCR, pmol O_2_/min) in primary brown adipocytes differentiated *in vitro* from BAT stromal vascular fraction of Ctrl and *Cdkal1* A-KO mice. Shown are average OCR from Ctrl (black) and A-KO (orange) primary brown adipocytes measured by Seahorse assay prior to stimulation and following injection of oligomycin (1 μM), FCCP (0.4 μM), and rotenone (3 μM) treatment. (B) Basal, leak, and maximal respirations of primary brown adipocytes are shown. (C) OCR from Ctrl and A-KO mitochondria isolated from BAT and measured by Seahorse assay under unstimulated conditions and after GDP (1 mM), oligomycin (14 μM), FCCP (10 μM), or rotenone (14 μM) treatment. (D) State IV, UCP1-independent, leak, and maximal respiration rates of isolated BAT mitochondria are shown. Error bars represent SEM. *, p < 0.05, **, p < 0.01, ***, p < 0.001, Student's *t*-test.

To test whether cellular metabolism was impaired in adipocytes in the absence of *Cdkal1*, we measured oxygen consumption in *ex vivo* differentiated primary brown adipocytes. Oxygen consumption rates (OCR) were significantly reduced in primary brown adipocytes from A-KO mice under basal, leak, and maximal respiration conditions (Figure 2A and B). To assess whether this defect could be localized to mitochondria *per se*, we isolated mitochondria from BAT and performed Seahorse respiration assays. While A-KO mitochondria demonstrated a higher basal (State IV) OCR, once GDP was added to inhibit Ucp1, we observed lower levels of Ucp1-independent respiration (Figure 2C and D). Similar decreased OCR levels were seen in mitochondria from A-KO cells for proton leak respiration, and for maximal respiration through the electron transport chain driven by the synthetic uncoupler FCCP (Figure 2C and D). These results suggest that loss of Cdkal1 affects mitochondrial function *per se*.

### 3.5 Aberrant mitochondrial morphology observed by EM in BAT from *Cdkal1* A-KO mice

**Figure 3:**
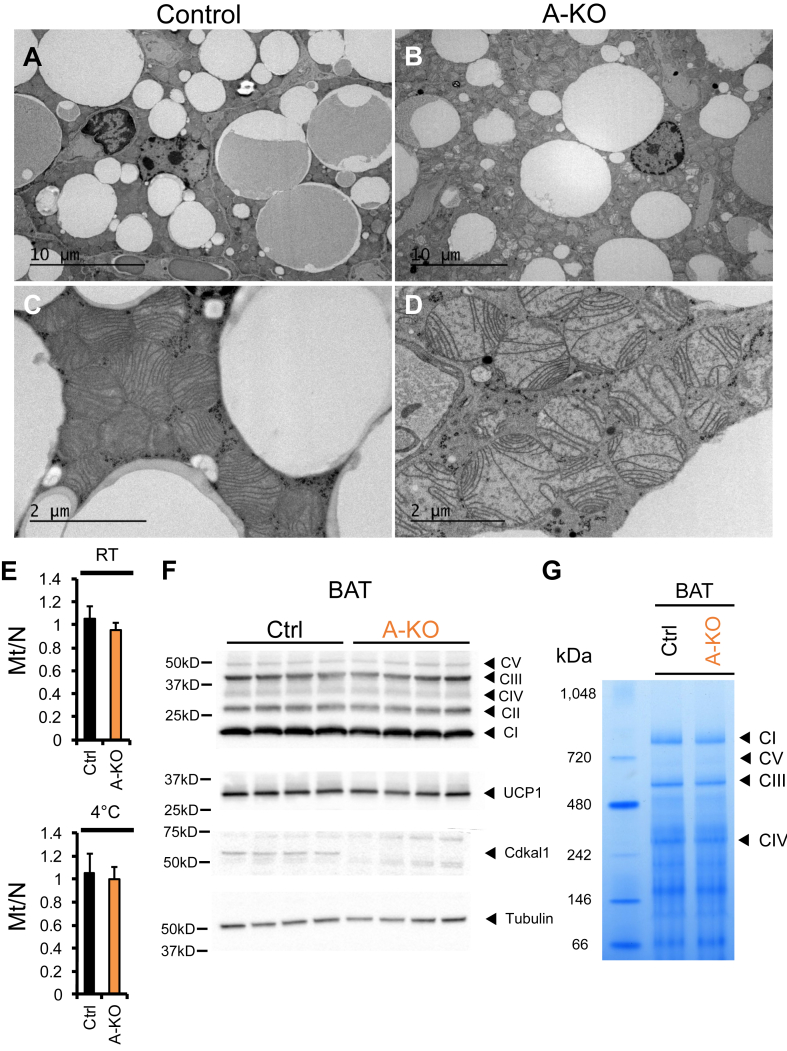
**Abnormal BAT mitochondrial morphology in Cdkal1 A-KO mice**. (A) Electron micrographs of BAT from chow-fed, room temperature housed control (A, C) or A-KO (B, D) mice. (E) Mitochondrial DNA to nuclear DNA ratio in BAT from mice housed at room temperature (RT) (Ctrl, n = 4; A-KO, n = 4) or subjected to 2.5 days of 4 °C cold temperature challenge (Ctrl, n = 5; A-KO, n = 6). (F) Western blot for protein levels of mitochondrial electron transport chain complex components (Mitoprofile), UCP1, and Cdkal1 in BAT of 26 week old, chow-fed Ctrl (n = 4) and A-KO (n = 4) male mice. Anti-β-tubulin blot was used to confirm equal protein loading. (G) Coomassie stain of Blue Native gel for mitochondrial lysate from Ctrl and *Cdkal1* A-KO BAT. Bands for native mitochondrial electron transport chain complexes are indicated.

Based on the decreased cellular and mitochondrial rates of respiration, we examined BAT cellular morphology by transmission electron microscopy. In contrast to BAT from control animals (Figure 3A and C), *Cdkal1* A-KO BAT mitochondria exhibit a swollen shape and disordered cristae distribution with a pale matrix (Figure 3B and D). In a medium-power field, dozens of abnormal mitochondria are apparent. Based on these findings, we sought to directly assess BAT mitochondrial number and quality. We quantified the ratio of mitochondrial to nuclear DNA in samples from mice housed at room temperature or following a 4 °C cold challenge. We observed no differences in mitochondrial DNA content in BAT of *Cdkal1* A-KO mice (Figure 3E). Western blotting for dissociated components of the electron transport chain complexes or the uncoupling protein UCP1 demonstrated no apparent differences in protein levels of these mitochondrial proteins in *Cdkal1* A-KO BAT (Figure 3F). Lastly, we also assessed relative levels of intact, native electron transport chain complexes from control and A-KO BAT mitochondria using non-denaturing Blue Native gels and Coomassie staining, observing modest decreases in complex I and III (Figure 3G). These findings of altered mitochondrial morphology further support a selective effect of Cdkal1 on mitochondria.

### 3.6 Total peptides levels and Lys codon utilization determined by mass spectrometry in *Cdkal1* A-KO white adipose tissue

**Figure 4:**
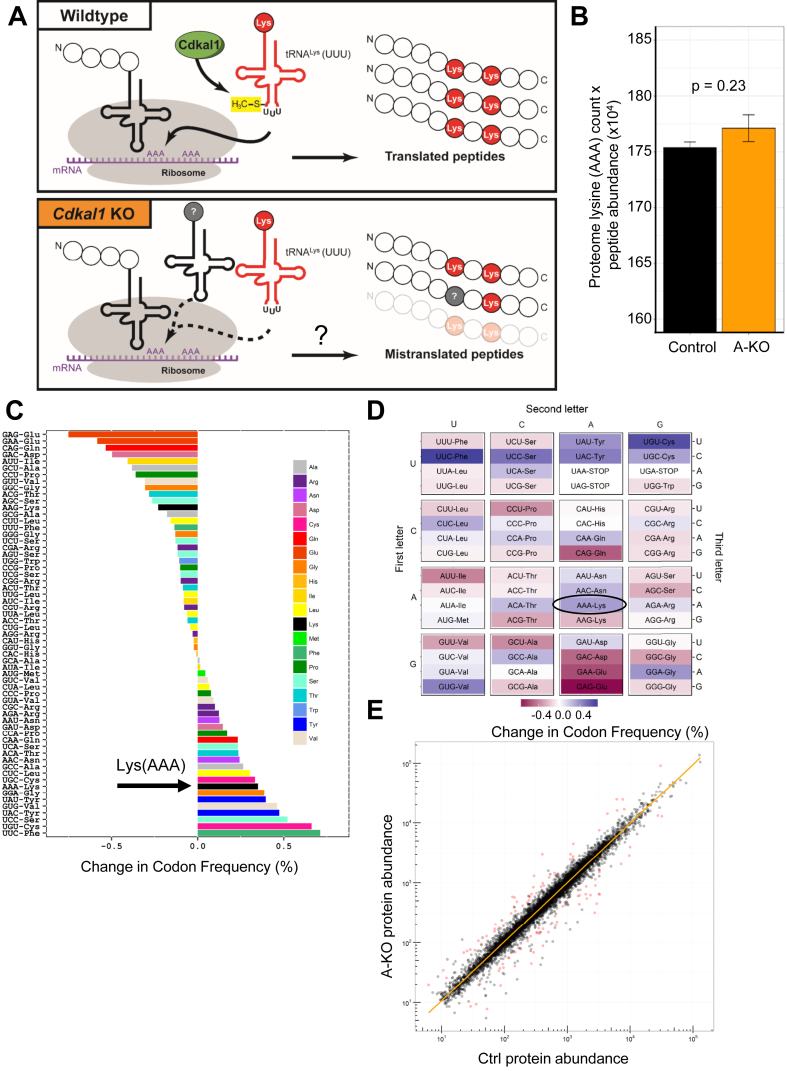
**Lys(AAA) codon usage in total peptides and downregulated peptides in Cdkal1 A-KO adipose tissue**. (A) Schematic depicting Cdkal1 function in methylthiolating t^6^A_37_ on tRNA^Lys^. Shown are expected effects on translation following *Cdkal1* knockout, including reduced Lys(AAA) codon translation fidelity and decreased proteins levels of derived from impaired translation of Lys(AAA)-rich coding sequences. (B) Lys(AAA) codons detected by TMT MS in visceral adipose tissue from chow-fed Ctrl (n = 5; black bar) and A-KO (n = 5; orange bar) mice. For each adipose sample, codon counts per peptide were multiplied by peptide abundance. Abundance-corrected codon counts were summed per mouse. Shown are averaged total proteome Lys(AAA) counts for each genotype. Error bars represent SEM. Mean Ctrl and A-KO Lys(AAA) codon counts were compared via Student's *t*-test. (C–D) Alterations in codon frequency (%) in peptides detected in A-KO visceral adipose tissue. Shown are a histogram (C) and codon heatmap (D) displaying enrichment or depletion in % codon frequency in peptides significantly downregulated by TMT-MS in A-KO visceral adipose tissue. (E) Log–log plot of proteins significantly upregulated or downregulated >2-fold (red circles) by TMT-MS in Cdkal1 A-KO visceral adipose tissue.

Based on the historical role of Cdkal1 in modifying tRNA^Lys^, we next tested whether reducing *Cdkal1* expression affects translation and global protein levels in adipocytes *in vivo*. To avoid confounding effects from examining BAT with abnormal mitochondria, we performed unbiased tandem mass tag (TMT) mass spectrometry to identify changes in total protein levels in visceral white adipose tissue (WAT) from chow-fed A-KO and control mice housed at room temperature. We detected 78,286 distinct peptides which correspond to 7,029 proteins. We hypothesized that loss of Cdkal1-dependent tRNA^Lys^ modification could increase levels of peptides mistranslated or degraded due to decreased fidelity of Lys incorporation (Figure 4A). To examine whether *Cdkal1* loss in adipocytes affects tRNA^Lys^ function, we examined Lys(AAA) residue counts in total peptides detected by TMT MS. We found that Lys(AAA)-encoded residues were not significantly decreased in the total proteome of A-KO adipose tissue (Figure 4B). We then tested whether proteins displaying reduced expression levels in *Cdkal1* A-KO adipose tissue were enriched preferentially for Lys(AAA) codons in their coding sequence. Comparing all codon frequencies (by percentage of each codon in the peptide coding sequence) in peptides >2-fold downregulated in A-KO adipose tissue versus total peptides, the Lys(AAA) codon ranks just eighth out of 61 codons for increased representation in downregulated peptides (Figure 4C). In addition, no significant differences were observed when codon utilization was calculated on only mitochondrial proteins (data not shown). Since Cdkal1 modifies t^6^A_37_, and its bacterial homolog MtaB can target t^6^A_37_ to regulate translation of all ANN codons, we also asked whether *Cdkal1* A-KO adipose tissue displayed altered frequency of ANN codons in downregulated peptides. We plotted changes in codon frequency by codon table, but no consistent or significant changes in ANN codon frequency emerged (Figure 4D). Regression analysis using a mixed effects model also detected no significant association between the percent Lys(AAA) codon frequency of a peptide and the fold change in expression levels of that peptide in A-KO visceral WAT (p = 0.198). Furthermore, although we predicted large effects on protein translation due to the role of Cdkal1 in regulating cytosolic tRNA^Lys^ modification and translation fidelity, we detected only modest changes in the proteome of visceral adipose tissue lacking Cdkal1. We observed fewer than 100 proteins meeting the criteria of >2-fold expression changes (Figure 4E). These data suggest that Lys codon translational fidelity was unaffected in adipose tissue lacking Cdkal1 expression.

### 3.7 Identification of ANT1 as a novel protein interactor of CDKAL1 by co-immunoprecipitation and mass spectrometry

**Figure 5:**
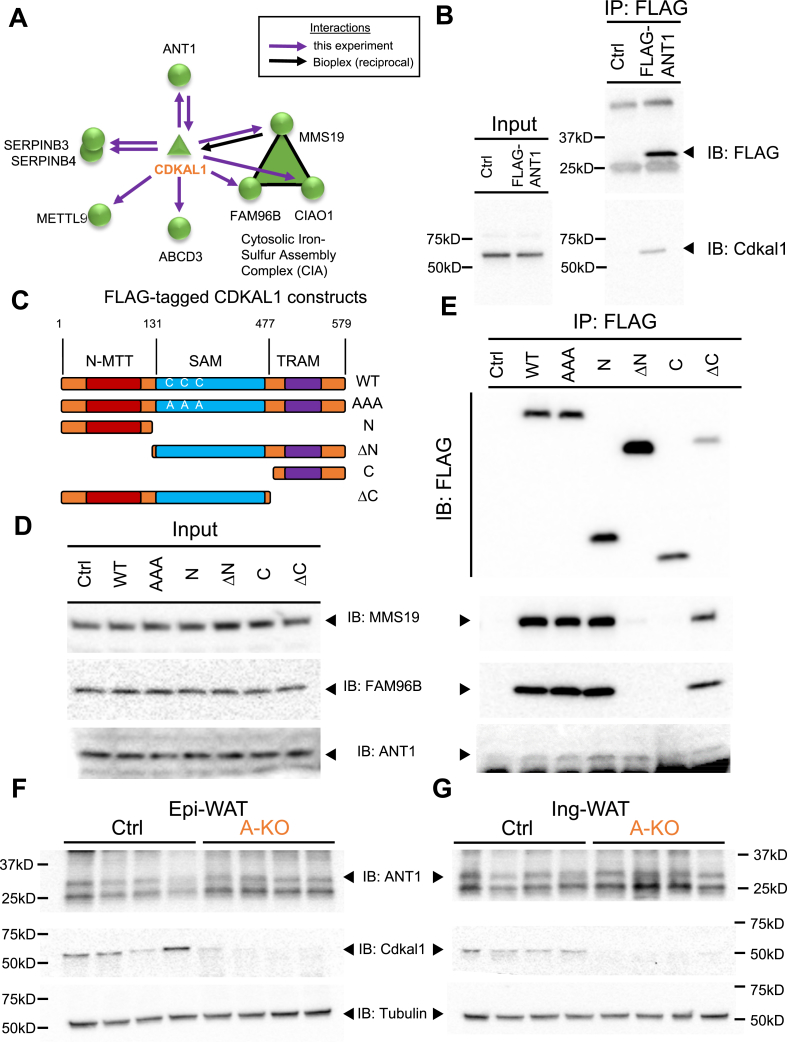
**Cdkal1 protein interacts with the cytosolic iron–sulfur cluster transfer complex proteins and the adenine nucleotide transporter ANT1**. (A) Proteins interacting with CDKAL1 as identified in this study by co-immunoprecipitation of FLAG-CDKAL1 followed by mass spectrometry (purple arrows). CDKAL1 protein interactors include components of the cytosolic Fe-S cluster transfer complex (MMS19, FAM96B, and CIAO1) and the mitochondrial protein ANT1. Reciprocal interaction of ANT1 with endogenous CDKAL1 is also represented. Also shown is the interaction of MMS19 with endogenous CDKAL1 from the BioPlex interactome project (black arrow) [25]. (B) Co-immunoprecipitation of endogenous CDKAL1 with FLAG-tagged ANT1. (C) Diagram of FLAG-tagged CDKAL1 mutants used in this study. Input (D) and (E) co-immunoprecipitation of FLAG-tagged wild type CDKAL1 or CDKAL1 mutants. Shown are immunoblots for FLAG-CDKAL1, MMS19, FAM96B, and ANT1 following anti-FLAG immunoprecipitation from lysates of HEK293 cells transfected with empty pcDNA 3.1 vector (Ctrl) or pcDNA 3.1 expressing: wild-type full-length CDKAL1 (WT); full-length CDKAL1 with radical SAM domain catalytic-site cysteines mutated to alanine (AAA); truncation mutants that include only (N) or lack (ΔN) an N-terminal fragment containing the UPF004 N-terminal MTT domain; and truncations mutants that comprise only (C) or lack (ΔC) a C-terminal fragment containing the TRAM domain. Protein levels of ANT1, CDKAL1 and β-tubulin in chow-fed control or A-KO eWAT (F) or iWAT (G).

To explore whether CDKAL1 may affect mitochondrial function through a novel biochemical function independent of tRNA^Lys^ modification, we mapped endogenous protein interactors of CDKAL1. FLAG-tagged CDKAL1 was immunoprecipitated from transfected HEK293 cells and subjected to mass spectrometry in concert with the high-throughput BioPlex protein interactome project [38]. We identified 8 high-confidence interactors (Figure 5A). The interactors with the highest Normalized Weighted D-Score (NWD-Score) across replicate experiments had previously been identified by reciprocal interactions which associated with endogenous Cdkal1 [41], [42]. These three proteins (MMS19, CIAO1, and FAM96B) are core components of a cytosolic iron–sulfur cluster assembly (CIA) complex that transfers iron–sulfur cluster cofactors onto cytosolic proteins, including CDKAL1 [39], [43]. Similarly, reciprocal interaction of endogenous CDKAL1 with immunoprecipitated MMS19 was observed in the BioPlex experiments [25], [38] (Figure 5A). We also identified novel interactors of CDKAL1, including mitochondrial protein ANT1/SLC25A4, an ATP/ADP transporter located in the inner mitochondrial membrane. Novel protein interactions can help to understand the biochemical function of CDKAL1, including a link between Cdkal1 deficiency and mitochondrial dysfunction via ANT1.

### 3.8 Mapping interaction domains of CDKAL1 with ANT1 and CIA complex proteins MMS19 and FAM96B

We confirmed the reciprocal interaction by immunoprecipitating FLAG-tagged SLC25A4/ANT1 and performed Western blotting for endogenous *Cdkal1* (Figure 5B). We next mapped the domains of CDKAL1 necessary for interaction with ANT1 and MMS19 (Figure 5C–E). We transfected constructs expressing full-length wild-type FLAG-CDKAL1 (WT) or a full-length CDKAL1 mutant that replaced three cysteine residues critical for [4Fe-4S] binding in the radical SAM domain with alanine (AAA), as well as constructs expressing truncation mutants containing only (N) or lacking (ΔN) the N terminal 131 amino acids encoding the conserved N-MTT domain, or containing only (C) or lacking (ΔC) the C-terminal residues 477–579 that comprise the TRAM domain (Figure 5C). Both MMS19 and FAM96B interact with WT, AAA, N, and ΔC fragments but not ΔN or C fragments (Figure 5D and E). These results suggest the N-MTT domain is necessary and that the conserved SAM domain cysteines and the C-terminal TRAM domain are dispensable for this interaction with a two core CIA proteins. ANT1 interacts with all tested fragments of CDKAL1, although much more weakly with the C-terminal TRAM domain alone (Figure 5E). We then tested whether Cdkal1 loss in adipose tissue positively or negatively affected protein levels of ANT1 *in vivo*. In epididymal and inguinal adipose tissue depots of A-KO mice, we observed increased protein levels of ANT1 in the absence of Cdkal1 protein (Figure 5F and G).

## 4 Discussion

The Cdkal1 locus is linked by GWAS to increased risk of developing the chronic diseases type 2 diabetes, ulcerative colitis, and psoriasis [44], [45], [46]. We found that obesity downregulates *Cdkal1* mRNA levels in mouse adipose tissue (Supplementary Figure 1D), spurring us to investigate whether adipocyte-specific loss of Cdkal1 can contribute to obesity-related metabolic disorders in mice on a high fat diet. Glucose tolerance and insulin sensitivity were unchanged in mice that lacked Cdkal1 in their adipocytes (Supplementary Figures 3 and 4). Instead, we observed decreased energy expenditure in Cdkal1 A-KO mice exposed to 4 °C cold-temperature challenge, suggesting altered respiration in brown or beige adipocytes *in vivo* (Figure 1D and E). Respiration was also reduced in Cdkal1 deficient primary brown adipocytes (Figure 2A and B) and isolated brown adipose mitochondria *in vitro* (Figure 2C and D). These results are concordant with defective mitochondrial function in adipocytes in the absence of Cdkal1.

Notably, the mitochondrial phenotype of *Cdkal1* A-KO mice shares features with that seen in mice with global deletion of the *Cdkal1* homolog, *Cdk5rap1*. *Cdk5rap1* KO mice exhibit defective mitochondria and impaired mitochondrial function in skeletal and cardiac muscle [40]. These effects were attributed to loss of modifications on mitochondrial tRNA species and subsequent mitochondrial translation impairment [40]. We demonstrate several lines of evidence that absence of Cdkal1 in adipocytes can affect mitochondrial morphology (Figure 3A–D) and function (Figure 2C and D). These adipose mitochondrial defects occurred independently of differences in body weight or glucose homeostasis (Supplementary Figures 3 and 4) or mitochondrial defects due to decreased Cdkal1 levels in other metabolically important tissues. Previous mouse studies using whole-body *Cdkal1* knockout demonstrated that lowered insulin secretion from islets lacking Cdkal1 coincided with reduced islet mitochondrial ATP production [20]. Together, our data support a model wherein perturbing Cdkal1 expression may produce effects on mitochondrial function in islets and other tissues like skeletal muscle, which may contribute to the pathogenesis of type 2 diabetes.

The accepted biochemical function of Cdkal1 protein is methylthiolation of specific tRNA species with the help of [4Fe-4S] cofactors, and *Cdkal1* deletion has been shown to block modification of murine tRNA^Lys^ (UUU) *in vivo* [18]. One proposed mechanism for how improperly modified tRNA^Lys^ causes impaired insulin secretion is via improper translation of critical lysines in pro-insulin, resulting in reduced pro-insulin levels [18]. We demonstrated that CDKAL1 interacts with MMS19, FAM96B, and CIAO1, core components of the cytosolic Fe-S cluster transfer machinery (Figure 5). Interaction with MMS19 is concordant with previously published reciprocal interaction with endogenous Cdkal1 [41], [42], and association with the CIA machinery likely provides Cdkal1 with Fe-S cluster cofactors needed for MTT enzymatic function. Surprisingly, however, we found no evidence in adipose tissue for defects in tRNA^Lys^ (UUU) function, as evaluated by Lys codon incorporation in total adipose peptides (Figure 4). Additionally, human ESC-derived CDKAL1^−/−^ β cells were recently shown to have decreased insulin secretion but not defects in translated insulin levels, consistent with an effect of Cdkal1 loss on insulin secretion that is distinct from regulating pro-insulin translation fidelity [10].

How Cdkal1 protein could regulate mitochondrial function remains unclear. As a cytoplasmic tRNA^Lys^ (UUU) modifier, Cdkal1 could be hypothesized to affected Lys incorporation and translation of mitochondrial proteins encoded in the nuclear genome, analogously to the mechanism by which Cdk5rap1 was proposed to regulate mitochondrial tRNA modification and mitochondrial translation [40]. However, we observed no evidence of Lys translation fidelity defects in total peptides from A-KO adipose tissue (Figure 4). To investigate novel pathways through which Cdkal1 could regulate mitochondrial function, we identified endogenous protein interactors (Figure 5A). We identified and confirmed reciprocal interactions between Cdkal1 and one such interacting protein Slc25a4/ANT1 (Figure 5A and B), the mitochondrial adenine nucleotide translocator which exchanges newly synthesized ATP into the cytoplasm and recycles ADP back into the mitochondria. As we observed with *Cdkal1* expression in mice (Supplementary Figure 1E), ANT1 is highly expressed in tissues rich in mitochondria, including heart, skeletal muscle, and BAT [47]. ANT1 is a regulator of mitochondria-mediated apoptosis, providing another link to cellular dysfunction [36]. ANT proteins also can function to uncouple mitochondria in brown adipose tissue. Previous work using the ANT protein inhibitor, carboxyactractyloside, has shown that blocking ANT function in BAT mitochondria impairs basal or leak respiration levels [48]. Reduced UCP1-independent and proton leak respiration in *Cdkal1* A-KO BAT mitochondria (Figure 2C and D) are thus consistent with effects on mitochondrial OCR expected from ANT1 disruption.

Strikingly, patients with missense mutations in ANT1 have been shown to develop disorders characterized by mitochondrial dysfunction and progressive weakening of their eye, heart, or skeletal muscles [49], [50]. Electron micrographs from human patients with ANT1 mutations reveal mitochondria that display a swollen, disorganized morphology [51], similar to that we observed in *Cdkal1* A-KO BAT mitochondria (Figure 3A–D). Patients and mice harboring defective alleles of ANT1 have impaired mitochondrial function and altered mitochondrial morphology [52], [53]. In addition, elevated levels of ANT1 are also observed under pathological conditions including facioscapulohumeral muscular dystrophy [54], [55], suggesting that inappropriately low or high levels of this protein can lead to mitochondrial disease pathogenesis. The *Cdkal1* A-KO mouse line, which exhibited abnormal mitochondrial morphology (Figure 3) and defective mitochondrial respiration (Figure 2C and D) in BAT, also exhibited increased levels of ANT1 in white adipose tissue (Figure 5F and G; Supplementary Figure 5). Understanding the mechanisms by which Cdkal1 may affect mitochondrial ANT1 levels could elucidate a novel mechanism by which Cdkal1 can regulate mitochondrial function.

Whether congenital defects in mitochondrial function are a significant contributor to the pathogenesis of common forms of diabetes is actively contested [56]. However, our data from *Cdkal1*-deficient adipose tissue suggest that dysregulation of mitochondria, in addition to impairment of Lys translation fidelity, is a cause for the diabetogenic effects of *Cdkal1* deletion *in vivo*. It remains to be tested whether the mitochondrial dysfunction we observed in adipose tissue from *Cdkal1* A-KO mice is mirrored in pancreatic islets or in other metabolically relevant tissues. Further understanding of whether genetic variation linked to type 2 diabetes within the CDKAL1 locus leads to decreased CDKAL1 expression and altered mitochondrial function in human patients will be an important next step in our investigations.

## Author contributions

CJP and ASB designed and performed experiments, analyzed data, and wrote the manuscript. RJB performed mass–spectrometry interaction mapping and assisted with manuscript preparation. JAP performed and analyzed TMT mass spectrometry experiments and assisted with manuscript preparation. Indirect calorimetry cold challenge was performed by AM, ZD and KBL. SPG, DEC, S. Hong, and JAH assisted in experimental interpretation and manuscript preparation. PHZ assisted in metabolic phenotyping experiments. S. Hagen and KS prepared BAT samples for electron microscopy, and generated and analyzed BAT electron micrographs. ASB is the guarantor of this work and, as such, had full access to all the data in the study and takes responsibility for the integrity of the data and the accuracy of the data analysis.

## Appendix A Supplementary data

**Supplementary Figure 1. Effect of obesity on adipose tissue expression of the type 2 diabetes GWAS gene *Cdkal1***. Type 2 diabetes-associated GWAS genes with decreased (A), increased (B), or unchanged (C) mRNA expression levels by qPCR in epididymal white adipose tissue (eWAT) from chow- versus HFD-fed C57BL/6J mice (n = 6 mice per group). (D) *Cdkal1* mRNA expression by qPCR in eWAT, inguinal WAT (iWAT), and liver from chow- versus HFD-fed mice (n = 5, chow; n = 6, HFD). (E) Levels of *Cdkal1* mRNA in tissues from chow-fed C57BL/6J mice (n = 6). Error bars represent SEM. Groups were compared by Student's *t*-test. *, p < 0.05; **, p < 0.01.

**Supplementary Figure 2. Mouse model for conditional deletion of Cdkal1**. (A) Schematic depicting the targeted Cdkal1 locus of the European Mutant Mouse Archive (EMMA) “null first” -to-conditional *Cdkal1* mouse strain (Cdkal1^tm2a(EUCOMM)Wtsi^), along with the cross strategy to *ACTB-FLP* deleter strain to generate conditional *Cdkal1*^flox^ mice with loxP-flanked exon 4. (B) Western blot probing for Cdkal1 protein in protein lysates from visceral eWAT depots of Ctrl and A-KO 17 week old male mice fed with normal chow diet. Anti-β tubulin blot was used to confirm uniform protein loading. (C) mRNA expression levels of Cdkal1 determined by qPCR in brown adipose tissue (BAT), epididymal WAT (eWAT), and subcutaneous inguinal WAT (iWAT) from 9 week old Ctrl (black bar) and A-KO (orange bar) male mice fed with standard chow diet *, p < 0.05; **, p < 0.01. Statistical comparisons were made using one-tailed Student's *t*-test.

**Supplementary Figure 3. Glucose homeostasis and insulin sensitivity in mice with adipose-specific Cdkal1 knockout on a standard chow diet**. (A) Blood glucose levels following IP-GTT on chow-fed A-KO male mice (n = 6) versus littermate controls (n = 7). (B) Blood glucose levels following ITT on chow-fed male A-KO mice (n = 5) versus littermate controls (n = 10). (C) Average body weights are shown at right. Error bars represent SEM. Statistical comparisons were made using one-tailed Student's *t*-test.

**Supplementary Figure 4. Body mass, body composition, glucose homeostasis, and insulin sensitivity in diet-induced obese mice with adipose-specific Cdkal1 knockout**. (A) Blood glucose levels following IP-GTT on 11–13 weeks HFD-fed Cdkal1 A-KO mice versus littermate controls. (B) Area under the curve (AUC) for GTT. (C) Blood glucose levels following ITT (represented as % initial blood glucose levels) from 12 to 15 weeks HFD-fed A-KO mice versus littermate controls (n = 16). (D) Average body weights at time of ITT. (E) Average body weights of male mice maintained on HFD for 14 weeks. (F) Body composition, represented as percent body fat mass and percent lean mass, in 10 weeks HFD-fed A-KO (n = 16 Control, n = 12 A-KO male mice for all panels). Error bars represent SEM. Statistical comparisons were made using one-tailed Student's *t*-test.

**Supplementary Figure 5. Schematic of Cdkal1 function**. (A) Schematic depicting modification of CDKAL1 by cytosolic iron–sulfur cluster assembly (CIA) complex proteins, MMS19, CIAO1, and FAM96B. The CIA complex binds to CDKAL1 and transfers dual 4Fe-4S clusters. CDKAL1 binds to and affects levels of ANT1, the mitochondrial ADP and ATP exchange protein. (B) Following CDKAL1 deletion, levels of ANT1 protein increase leading to abnormal mitochondrial morphology and function.
