# Supplementary material for: Cdkal1, a type 2 diabetes susceptibility gene, regulates mitochondrial function in adipose tissue
Source: Mol Metab. 2017 Jul 31;6(10):1212–25. doi: 10.1016/j.molmet.2017.07.013 (PMC5641635; doi:10.1016/j.molmet.2017.07.013)
Supplement: Supplementary file 2 [file mmc2.pdf]

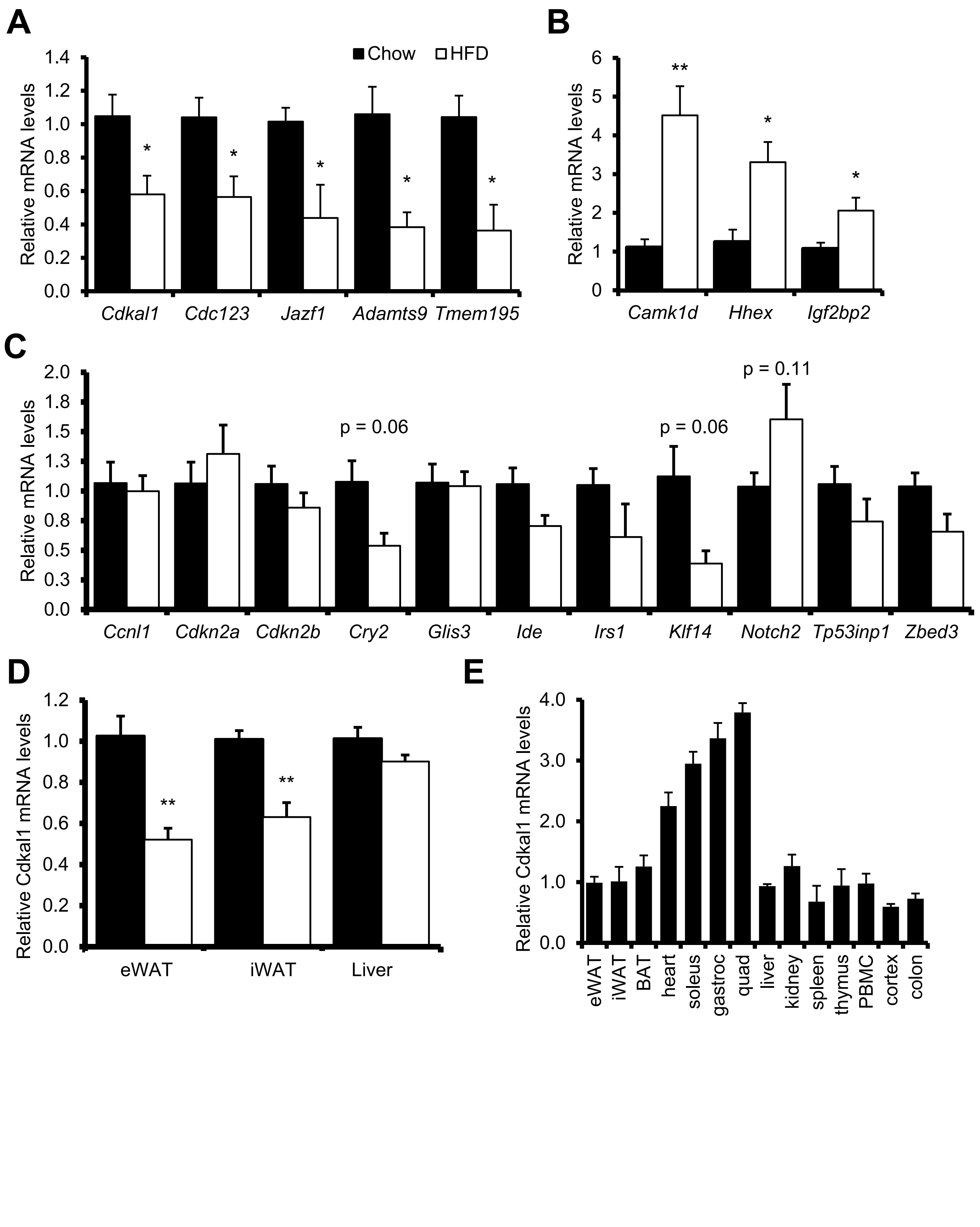

Supplemental Figure 1

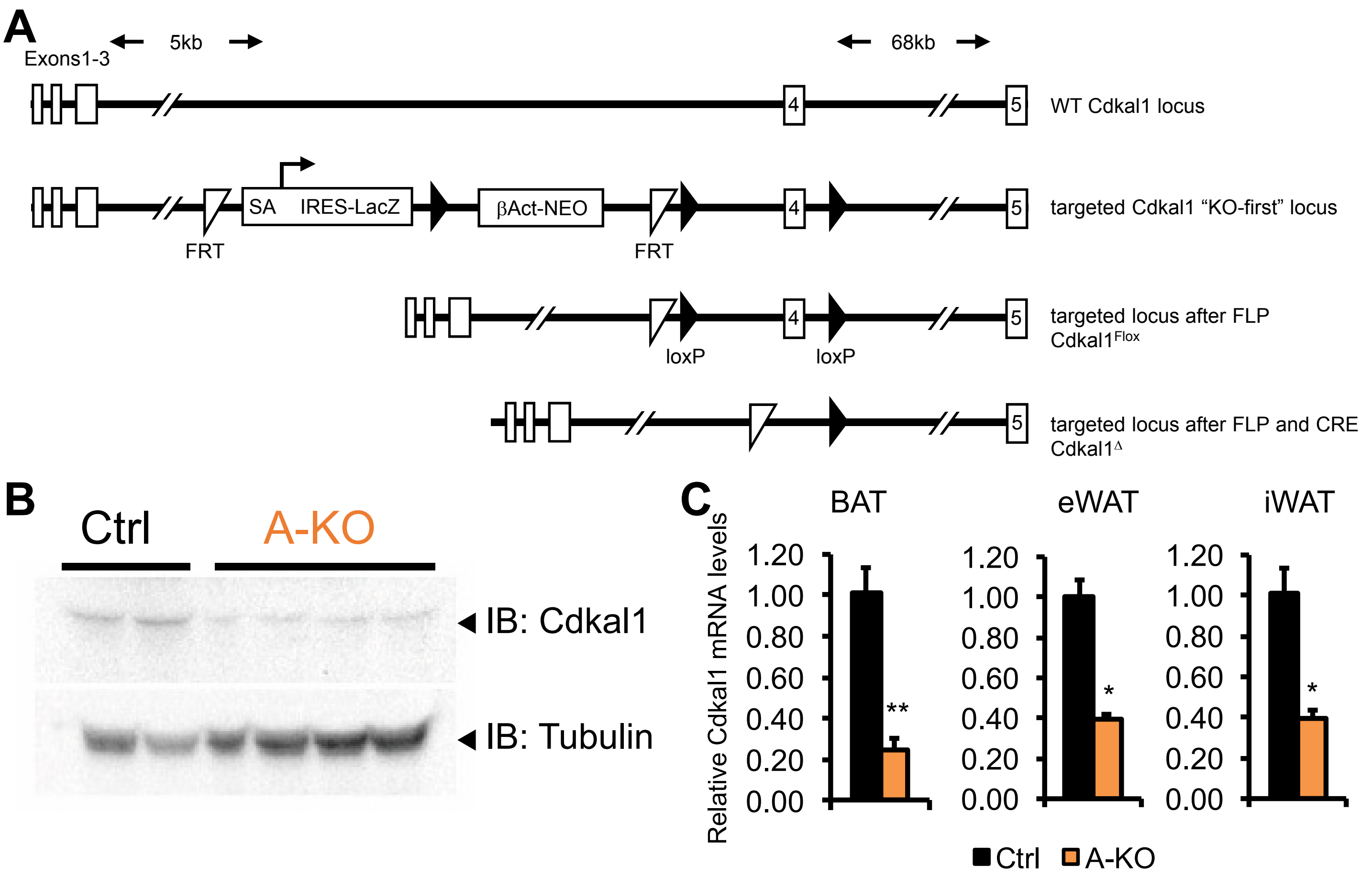

Supplemental Fig 2

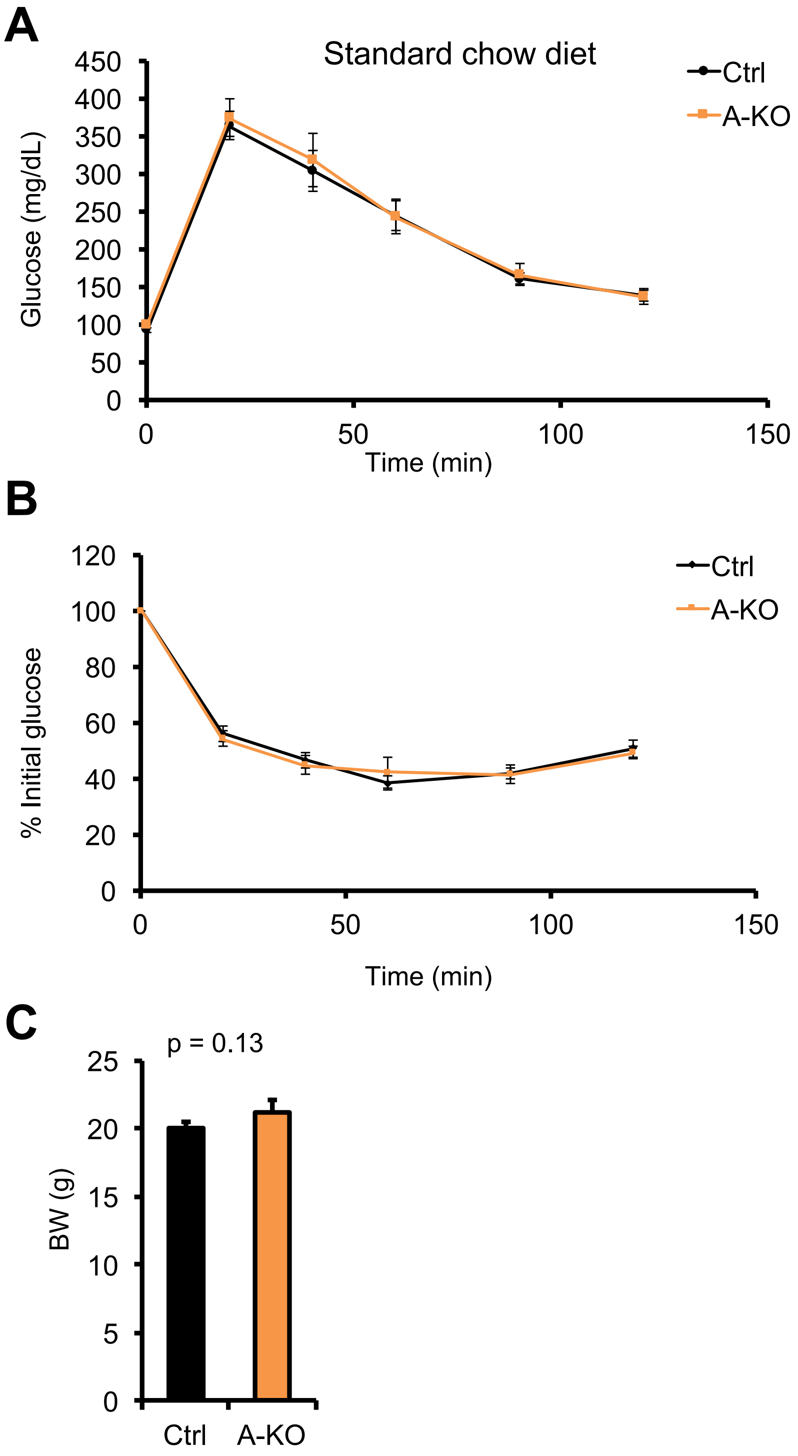

Supplemental Fig 3

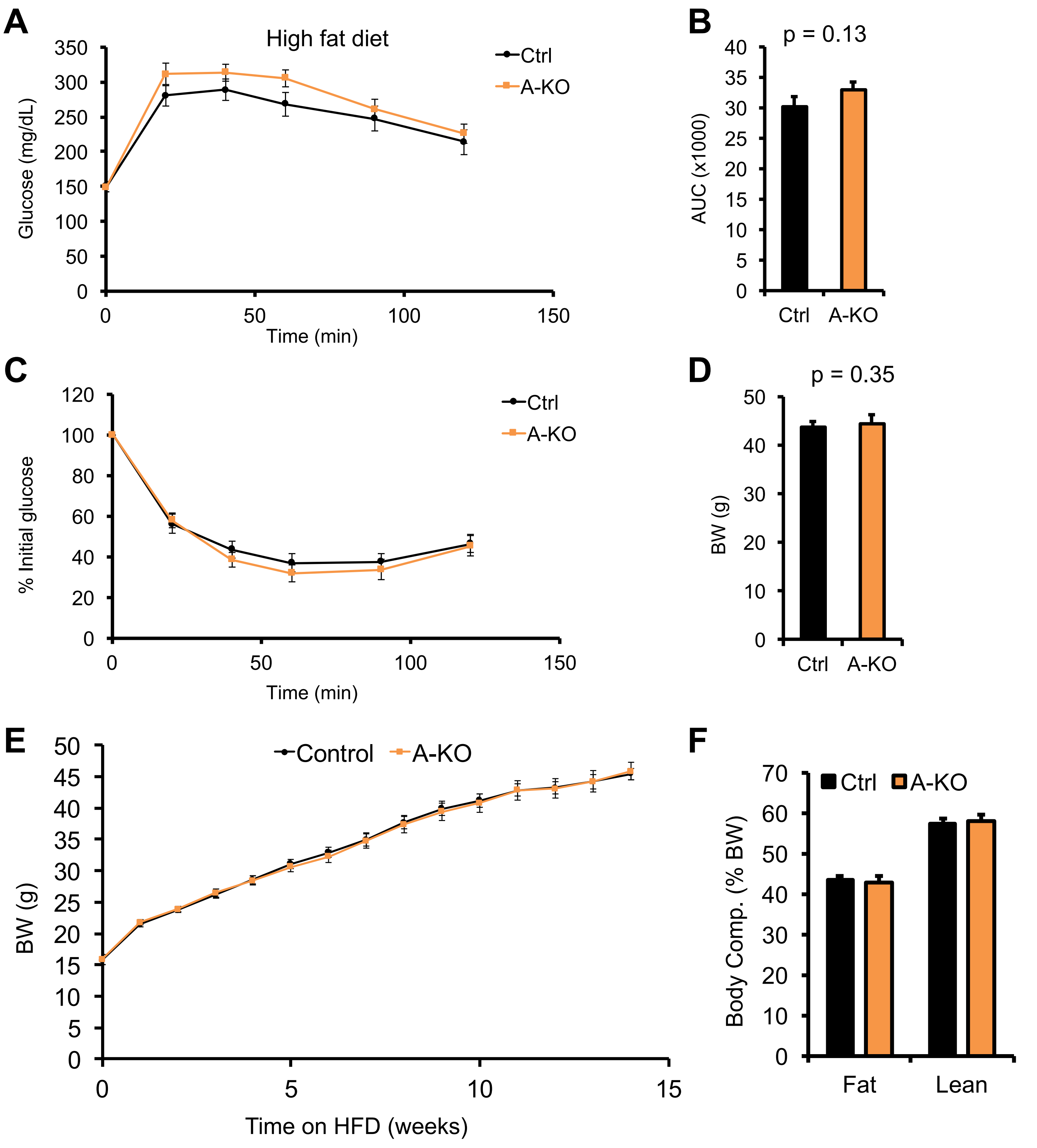

Supplemental Fig 4

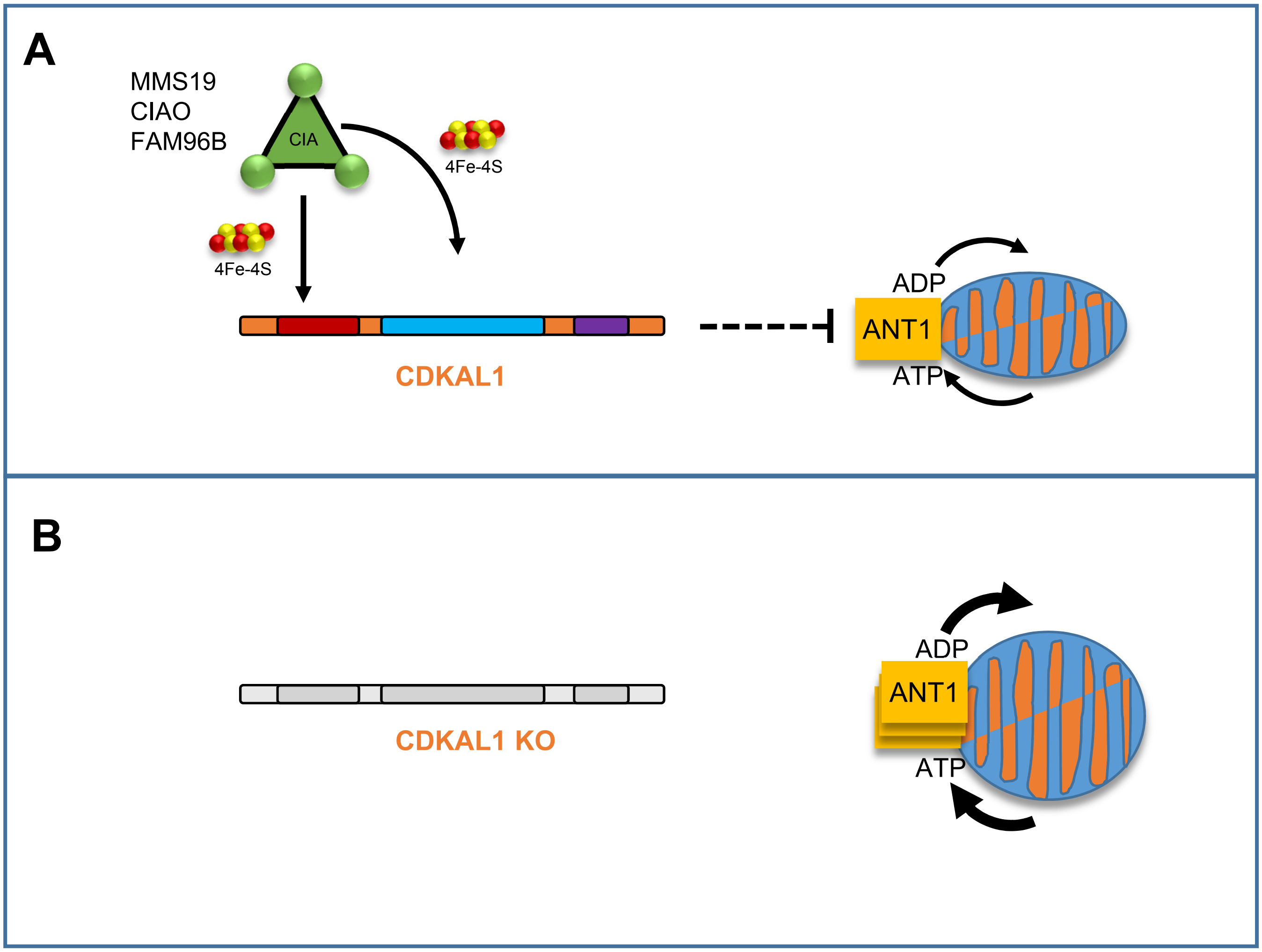

Supplemental Figure 5
